# A network based approach to drug repositioning identifies plausible candidates for breast cancer and prostate cancer

**DOI:** 10.1186/s12920-016-0212-7

**Published:** 2016-07-30

**Authors:** Hsiao-Rong Chen, David H. Sherr, Zhenjun Hu, Charles DeLisi

**Affiliations:** 1 Bioinformatics Program, College of Engineering, Boston University, Boston, MA USA; 2 Graduate Program in Translational Molecular Medicine, Boston University School of Medicine, Boston, MA USA; 3 Department of Environmental Health, Boston University School of Public Health, Boston, MA USA; 4 Department of Biomedical Engineering, Boston University, Boston, MA USA

**Keywords:** Computational drug repositioning, Drug screening, Cancer treatment

## Abstract

**Background:**

The high cost and the long time required to bring drugs into commerce is driving efforts to repurpose FDA approved drugs—to find new uses for which they weren’t intended, and to thereby reduce the overall cost of commercialization, and shorten the lag between drug discovery and availability. We report on the development, testing and application of a promising new approach to repositioning.

**Methods:**

Our approach is based on mining a human functional linkage network for inversely correlated modules of drug and disease gene targets. The method takes account of multiple information sources, including gene mutation, gene expression, and functional connectivity and proximity of within module genes.

**Results:**

The method was used to identify candidates for treating breast and prostate cancer. We found that (i) the recall rate for FDA approved drugs for breast (prostate) cancer is 20/20 (10/11), while the rates for drugs in clinical trials were 131/154 and 82/106; (ii) the ROC/AUC performance substantially exceeds that of comparable methods; (iii) preliminary in vitro studies indicate that 5/5 candidates have therapeutic indices superior to that of Doxorubicin in MCF7 and SUM149 cancer cell lines. We briefly discuss the biological plausibility of the candidates at a molecular level in the context of the biological processes that they mediate.

**Conclusions:**

Our method appears to offer promise for the identification of multi-targeted drug candidates that can correct aberrant cellular functions. In particular the computational performance exceeded that of other CMap-based methods, and in vitro experiments indicate that 5/5 candidates have therapeutic indices superior to that of Doxorubicin in MCF7 and SUM149 cancer cell lines. The approach has the potential to provide a more efficient drug discovery pipeline.

**Electronic supplementary material:**

The online version of this article (doi:10.1186/s12920-016-0212-7) contains supplementary material, which is available to authorized users.

## Background

The high cost and the long time required to bring drugs into commerce [1–3] is driving efforts to repurpose FDA approved drugs—to find new uses for which they weren’t intended, and to thereby reduce the overall cost of commercialization, and shorten the lag between drug discovery and availability [4]. Among the successes of this approach are sildenafil, originally developed as a cardiovascular drug [5] and repositioned to treat erectile dysfunction; and zidovudine (AZT), originally developed as an anticancer drug [6], and repositioned for the treatment of HIV. These discoveries, though serendipitous, motivated more systematic approaches which might amplify the number of discoveries many-fold.

Systematic approaches generally begin with some form of computer based screening to generate large numbers of plausible candidates [7–11]. Many current computational strategies exploit shared similarities among drugs or diseases and infer similar therapeutic applications or drug selections. Drug similarities include chemical structures [12–14], drug-induced phenotypic side effects [12, 15], molecular activities [16]. Disease similarities include phenotypic similarity constructed by identifying similarity between MeSH terms [17] from OMIM database [18]; semantic phenotypic similarity [12]. The efficacy of the candidates generated by such approaches would not exceed that of existing drugs since the disease biomarkers remain the same.

A more general approach searches for disease (Gene Expression Omnibus, GEO) and drug (CMap) induced transcriptional profiles that are inversely correlated [19–23]. Strong anti correlation between the gene expression profiles of an FDA approved drug and those of a disease for which it was not intended identifies the drug as a candidate for repositioning. This procedure, though useful, is relatively agnostic with respect to the functional relations between profiles (the ordered lists of perturbed genes). A drug identified this way is limited in that it is not informed by cellular function, but simply targets a group of generally non-interacting differentially expressed genes.

The idea underlying our method, which we refer to as the method of functional modules (MFM), is to impose the condition that candidates must affect the *same cellular functions* in opposite ways, and to use information about DNA as well as RNA. In particular we search for drugs that strongly perturb sets of genes having the following properties: (i) they share a strong functional relationship (ii) they are mutated in the disease state (iii) their expression is highly perturbed by the disease (iv) they are within significantly perturbed pathways of diseases. Functional association is based on position in a human functional linkage network (FLN) [24]—an evidence weighted network that provides a quantitative measure of the degree of functional association among any set of human genes. This means the method integrates multiple sources of evidence such as protein-protein interactions and is not limited to catalogued functional associations, e.g. KEGG, but uses a general approach to find functional modules.

We used genome-wide transcriptional data for more than 3500 compounds provided by LINCS [25] and identified 519 (410) repositioned drug candidates for breast (prostate) cancer. We also compared the accuracy of our method with that of comparable approaches [20, 22] (see Results). We applied CMap datasets and ranked bioactive compounds using different methods, then compared the predictability of the ranked lists of compounds (see Statistical validation). We then presented evidence that a set of disease mutated genes and their nearest FLN neighbors (mutation associated genes (MAGs), see Methods) provided more functional insight than a set of differentially expressed genes in the disease.

In addition to these computational assessments, in vitro viability tests confirmed that 4 our predicted drug candidates were more efficacious than Doxorubicin--an FDA-approved drug for breast cancer--against MCF7 and SUM149 cell lines.

## Methods

The method built non-incrementally on the work of Shigemizu et al. [22]. In particular: (i) we took account of information on mutations (DNA) as opposed to just expression (RNA); and (ii) we took account of functional information by using a so-called FLN [24], as explained below. Specifically, we annotated mutated genes on the FLN [24], and identified and eliminated all genes that 1) are not within a specified distance of a mutated gene (the functional module constraint); 2) have a differential expression below some threshold (the disease condition constraint); 3) are not in pathways that distinguish the cancer/normal phenotype.

An FLN [24] is represented as a network of nodes (genes/proteins) connected by links whose weights are proportional to the likelihood that the connected nodes share common biological functions. We set a threshold on linkage weight so as to exclude approximately 95 % of the neighbors of any given node, leaving clusters of functionally related aberrant genes. We carried out the procedure twice, once starting with mutated genes and their first nearest neighbors, and then with mutated genes and their first and second nearest neighbors.

**Fig. 1:**
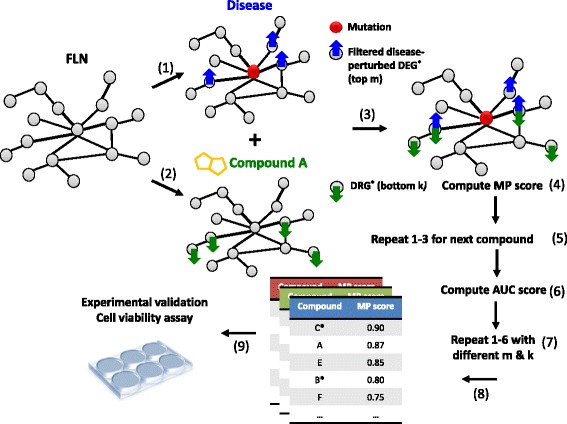
Analytic workflow. (1) After mapping mutated genes to the FLN, identify the functional neighbors that are up or down regulated (DEG: differentially expressed genes) and within significantly enriched disease pathways (FDR < 0.05). (2) Map the genes that are down or up regulated by drug candidates to the FLN (3) Compute the MP score; i.e. the significance of the functional overlap between the drug and disease perturbed genes (see text). (4) Rank the compounds according to the MP score. (5) Compute the sensitivity and specificity of the ranked list of compounds. (6) Repeat the process with different groups of MAG and DRG (Drug Response Gene) generated by looping over the parameters (m & k). (7) Choose the parameter set that has highest sensitivity and specificity. (8) The drug candidates are chosen form the ranked list generated by the best parameter set. (9) The top ranked drug candidates are chosen for in vitro experimental validation

We considered each drug in turn and identified two FLN landscapes: one defined by genes that are up-regulated by the disease and down regulated by the drugs (Up regulated Cancer gene, Down regulated Bioactive target gene--UCDB) and, the other defined by genes that are down regulated by disease and up regulated by the drug (DCUB). Each landscape was thus an interconnected set of drug and disease perturbed genes. Finally we assigned a score, mutual predictability (discussed below), which measured the *connectivity* within each landscape, which is roughly speaking the extent to which the drug and disease genes sets are correlated. The greater the relationship, the higher the likelihood that the drug is a viable candidate for repositioning. The methodology is summarized in Fig. 1. The specifics follow.

### Data sources

Well-documented mutated genes were downloaded from the Online Mendelian Inheritance in Man (OMIM) (http://www.ncbi.nlm.nih.gov/omim) [18]. 40 breast cancer and prostate cancer and 69 leukemia well-documented genes were obtained from OMIM (see Additional file 1). FLN was downloaded from http://visant.bu.edu/misi/fln/.

### Transcript levels

The differentially expressed genes were obtained from the Illumina HiSeq 2000 RNA Sequencing platform for 108 breast and 51 prostate paired tumor and normal samples, downloaded from the TCGA portal (http://cancergenome.nih.gov/). Differential expression data in response to leukemia (GSE1159, GSE9476) were obtained from the National Center for Biotechnology Information (NCBI) Gene Expression Omnibus (GEO) (http://www.ncbi.nlm.nih.gov/geo/). The ranked list of differentially expressed genes was generated using edgeR [26] and a t-statistic.

Ranked list of differentially expressed genes in response to compounds treated in breast cancer (MCF7 cell line), myelogenous leukemia (HL60 cell line), and prostate cancer (PC3 cell line) were obtained from connectivity map (CMap) build 02 [20], https://www.broadinstitute.org/cmap) and LINCS (level 4) (http://www.lincscloud.org/) [20].

### Mutation-associated genes (MAG)

The procedure maps to the FLN, known mutated drivers for the disease of interest, and their first nearest neighbors. It then sets the linkage threshold to 0.2, eliminating 95 % of the links and leaving gene clusters each of which is relatively homogeneous functionally. The remaining genes are further selected by 1) setting a threshold on transcription level; 2) filtering out the genes that are not in pathways that distinguish phenotype (i.e. cancer from normal--see Pathway enrichment analysis). As indicated below we were left with relatively small gene sets at the end of the process. In order to identify well-correlated drug-disease gene sets, the definitions of up- and down-regulated genes were not tightly constrained. In particular, we looped through *m* sets of various sizes, ranging from the 1000 most up-regulated genes, to the top half of the total number of genes in our universe--which depends on the number of probes on the chip--in increments of 2,000. A similar procedure was followed to obtain networks of the most down-regulated genes.

Networks were obtained for each member of our universe of bioactive compounds. A drug was ranked in accord with the intersection between its functional network and the disease functional network, as described below. The procedure was then repeated, by starting with first and second nearest neighbors. The final number of MAG ranged from 75 to 1074 for breast cancer; 15 to 460 for prostate cancer; and 46 to 772 for leukemia.

### Pathway enrichment analysis

We focused on the enrichment of pathways abnormally perturbed in the disease state compared to the normal state. PWEA [27] (http://zlab.bu.edu/PWEA/download.php) was used to identify significantly perturbed pathways in the gene expression profiles of breast cancer, leukemia and prostate cancer described above.

### Drug response genes (DRG)

The top (up-regulated) and bottom (down-regulated) *k* most differentially expressed genes in response to bioactive compounds in disease cell lines were selected as DRG. We restricted the number of up (down)-regulated DRG to be within +/− 500 genes of the matched down (up)-regulated MAG. For example, if 500 up-regulated MAG are in an FLN cluster, *k* would from a low of 100 to a high of 1000 in increments of 100.

### Library of Integrated Cellular Signatures (LINCS)

LINCS profiles are generated using 3,678 and 4,228 bioactive compounds for breast cancer and prostate cancer, respectively, each compound typically applied at 6 different concentrations (0.0003-177 μM) and 2 time points (6 and 24 h). We retained the expression profile of a compound that produced maximal mutual predictability score before ranking the compounds. Twenty of the 3678 (11 of 4228) were FDA approved drugs for breast (prostate) cancer.

### Connectivity map

We used CMap datasets for comparing the performance between our method with others. CMap profiles are generated using 1251, 1079 and 1182 bioactive compounds for breast cancer, leukemia and prostate cancer, respectively. Eight of the 1251, 6 of 1079, and 7 of 1182 were FDA approved drugs for breast cancer, leukemia and prostate cancer respectively.

### Drug and clinical trial information retrieval

We collected data from DrugBank (http://www.drugbank.ca/). FDA approved drugs from FDA service: Drugs@FDA. Clinical trial data were downloaded from https://clinicaltrials.gov.

### Mutual predictability (MP)

We used mutual predictability [4] to score the correlation between mutation associated genes (MAG) and drug response genes (DRG). In essence, mutual predictability is a measure of the degree to which MAG can be used as seed genes to predict DRG (predictability M-D), and vice versa (predictability D-M). The mutual predictability of the two sets measures the extent to which genes in one set can be used to identify (predict) genes in the other [24]. A disease drug pair with high mutual predictability has a strong functional relation; the higher the score, the stronger the relation.

To quantify the predictability M-D, we use MAG as seeds, and score and rank each gene connected to a seed using the disease mutual predictability score *S*_*i*_:

$$ {S}_i={\displaystyle \sum_{j\in seeds}}{w}_{ij} $$

where w_*ij*_ weights the link between gene *i* and seed *j, and* the score is 0 if there is no seed connection.

We obtained the sensitivity and specify variation by using a series of cutoffs on the ranked list. The number of true positives is taken to be the number of DRG above a particular cutoff; the number of true negatives is the number of non-DRG below the cutoff; the number of false positives is the number of non-DRG above the cutoff, and the false negatives are the number of DRG below the cutoff. AUC scores range from 0 and 1, with 0.5 and 1.0 indicating random and perfect predictive performance, respectively.

AUC_D-M_ as a measure of predictability D-M is similarly calculated. The mutual predictability between MAG and DRG is then defined as the geometric mean of AUC_D-M_ and AUC_M-D_:

$$ \mathrm{Mutual}\ \mathrm{Predictability}\ \left(\mathrm{M}\mathrm{A}\mathrm{G}\ \mathrm{and}\ \mathrm{D}\mathrm{R}\mathrm{G}\right) = \sqrt{{\mathrm{AUC}}_{\mathrm{D}-\mathrm{M}} \times {\mathrm{AUC}}_{\mathrm{M}-\mathrm{D}}} $$

Each bioactive compound is thereby ranked by its mutual predictability score.

A detailed example of MP score computation is shown in Additional file 2, 2-1 and Additional file 3 Figure S1.

### Evaluation of predictability

#### Statistical validation

We determined the extent to which FDA approved cancer drugs were enriched in our ranked list by again calculating an AUC as indicated above. Briefly, focus on a position *t* from the top. The ratio of FDA approved drugs for target disease at or above position *t*, to total drugs at or above *t* is counted as TP; the ratio of non-FDA approved drugs below *t* to total drugs below *t* is TN. The running index *t* is varied to produce a ROC, and the area under the curve (AUC) is used as a measure of predictability. This is of course a non-normalized result, but as we now indicate it is used only in a relative way, to compare different parameter sets.

### Parameter optimization

Each set of parameters (rank cutoffs *m & k* for filtering MAG and selecting DRG) generated different ranked lists of bioactive compounds. We computed the AUC score using the ranked list, and chose the best set of parameters based on the maximum AUC score. Repositioned drug candidates were selected from the ranked list generated by the best parameter set. After optimization, the best parameters (number of MAG and DRG (MAG/DRG)) are 237/700 (UCDB) and 75/100 (DCUB) for breast cancer; and 333/100 (UCDB) and 46/100 (DCUB) for prostate cancer.

For the ranked list, the significance of the mutual predictability scores for each compound was estimated by randomly selecting a set of n DRG, computing the mutual predictability score given the MAG, repeating the process 100,000 times to generate a null distribution, and then estimating the probability that our observation was obtained by chance. We computed the false discovery rate (FDR) for individual compounds by calculating the expected number of false positives, given the actual distribution of mutual predictability scores and the null distribution.

We assessed the significance of the best AUC score by randomly selecting from LINCS, 20 out of 3678 drugs for breast cancer and 11 out of 4228 for prostate cancer as true positives. For CMap, we randomly selected 8 out of 1251 drugs for breast cancer; 6 out of 1079 for leukemia; and 7 out of 1182 for prostate cancer. We then computed the AUC for each parameter set, repeated the process 100,000 times and generated a null distribution. The *p*-value was used to estimate FDR for multiple tests.

### Comparison with other methods

We applied the methods (Lamb et al. and Shegemizu et al.) that used CMap data to breast cancer, leukemia and prostate cancer and compared them with MFM.

### Lamb et al. [20]

We queried the 50 to 500 (in increments of 50) up- and down-regulated signature genes of breast cancer (MCF7), leukemia (HL60) and prostate cancer (PC3) on (https://www.broadinstitute.org/cmap/newQuery?servletAction=querySetup&queryType=quick), and obtained ranked lists of bioactive compounds. The disease signature genes (FDR < 0.05) were generated from the same expression data used for MFM, as described in Transcript levels. The total number of compounds and the corresponding cell lines were the same as those were used for MFM. Then we followed the same procedure as that was used for MFM to assess the performance. The highest AUC score was selected for comparison.

### Shegemizu et al. [22]

We used the same expression profiles (GDS2617, GDS2908 and GDS1439) and parameters (1200 and 1400 for UCDB and DCUB for breast cancer; 700 and 800 for UCDB and DCUB for leukemia; 5200 and 4200 for UCDB and DCUB for prostate cancer) reported in the [22] to generate ranked lists of compounds. Performance was assessed with the same procedure used for MFM.

### Experimental validation

#### Cell cultures and reagents

Cell lines MCF7, SUM149 and MCF10A were obtained from ATCC (American Type Culture Collection, Manassas, VA) and maintained as recommended. The growth medium was supplemented with 10 % fetal bovine serum (FBS), 50 units/ml of penicillin and streptomycin, and incubated at 37 °C with 5 % carbon dioxide. Dimethyl sulfoxide (DMSO), at 0.2 %, was used as the vehicle control.

### MTT assay

Metabolic activity of MCF7, MCF10A and SUM149 cells treated with vehicle (0.1 % DMSO) or repositioned drug candidates was assessed with the MTT (3-(4,5-dimethylthiazol-2-yl)-2,5-diphenyl tetrazolium bromide) assay. Cells were placed in 96-well plates and treated for 24 h with drugs with concentrations ranging from 0–1000 μM, then assayed for metabolic activity. 10 μl of MTT solution (10 mg/ml in PBS) was added to each well and incubated for an additional 3 h. The medium was then replaced with 200 μl of DMSO. Absorbance was determined at 570 nm (experimental absorbance and 690 nm (background absorbance) by an ELISA plate reader. The inhibitory effect of drug candidates was expressed as the relative metabolic activity (% control) and calculated as shown below. The relative viability was calculated as relative viability = (experimental absorbance - background absorbance)/ (absorbance of vehicle controls - background absorbance of vehicle controls) × 100 %.

## Results

We screened repositioned drug candidates by using mutual predictability [24] to score correlation between mutation-associated genes up-regulated in disease samples and genes down-regulated by bioactive compounds (DCUB), and vice versa (UCDB). Since a high mutual predictability score indicates strong functional linkage between sets of disease and drug related genes, our hypothesis is that candidate drugs so identified have potential to correct the sets of disease genes and have therapeutic effect on the disease.

### Identification of repositioned drug candidates for breast cancer and prostate cancer using LINCS

We performed analysis on the most updated data of gene expression signatures of bioactive compounds from LINCS [25]. We evaluated the significance of mutual predictability score of each compound, and FDRs as explained under Methods.

### Statistics of significant bioactive compounds

#### Breast cancer

LINCS includes breast cancer cell line expression in response to 3678 compounds. We calculated the mutual predictability score for each of these, as described in Method – Mutual Predictability Score. The gene sets associated with each cancer/compound were assigned *p*-values as described in Method – Parameter optimization, to obtain ranked lists of 2435 DCUB compounds and 1875 UCDB compounds with FDR < 0.05 (Table 1). Of these 510 were FDA approved drug candidates for repositioning to breast cancer. The detailed description of candidates is in Additional file 4.

**Table 1:** Breast cancer and prostate cancer repositioned drug candidates identified from analysis of LINCS. Complete lists of repositioned drug candidates for breast cancer and prostate cancer are shown in Additional file 13

	Breast Cancer		Prostate Cancer	
Total compounds	3678		4228	
Compounds that are FDA drugs	632		676	
Compounds that are FDA drugs for target disease	20		11	
Compounds that are in clinical trial for target disease	154		106	
	UCDB	DCUB	UCDB	DCUB
Compounds with FDR < 0.05	2435	1875	2500	1668
Compounds that are clinical drugs with FDR < 0.05 (*p*-value)	131 (6.2E-8)	109 (2.7E-7)	82 (4.9E-5)	67 (4.8E-7)
FDA drugs with FDR < 0.05	427	325	456	317
FDA drugs with FDR < 0.05 in both UCDB and DCUB	244		291	
FDA drugs for target disease with FDR < 0.05 (*p*-value)	20 (2.5E-4)	19 (2.7E-5)	10 (2.6E-2)	9 (5.3E-3)
AUC (*p*-value)	0.86 (<1.0E-6)	0.81 (<1.0E-6)	0.77 (9E-3)	0.83 (4.7E-5)
Number of MAG/DRG	237/700	75/100	333/100	46/100

**Table 2:** ^a^Mutual predicatbility score of breast cancer drug candiates predicted by MFM

FDA Drug	^a^MP score	*P*-value	FDR
Clotrimazole	0.7	5.00E-06	4.88E-05
Triprolidine	0.69	2.00E-05	1.64E-04
Thioridazine	0.69	2.00E-05	1.64E-04
Mefloquine	0.69	3.00E-05	2.28E-04
Fluphenazine	0.66	1.11E-02	2.13E-02

#### Prostate cancer

LINCS includes prostate cancer cell line expression in response to 4228 compounds. The gene sets associated with each cancer/compound were assigned *p*-values to obtain ranked lists of 2500 DCUB compounds and 1668 UCDB compounds with FDR < 0.05 (Table 1). Of these 291 were FDA approved drug candidates for repositioning to prostate cancer (Additional file 4).

### Supporting evidence

#### Sensitivity and specificity

To evaluate the predictability of the ranked drug candidates, ROC curves were generated using 20 FDA breast cancer drugs and 11 FDA prostate cancer drugs as true positive. The highest AUC scores were 0.86 (*p* = 1.0E-6) and 0.83 (*p* = 4.5E-5) for breast cancer and prostate cancer, respectively. We estimated the significance of the AUC scores as described in Parameter optimization session.

### Comparisons with computational drug repositioning methods

**Fig. 2:**
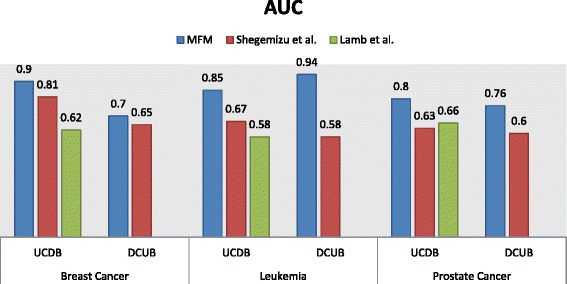
Comparison of performance for the MFM with other methods. We applied CMap datasets to compare performance of MFM with Shegemizu et al. and Lamb et al. The sensitivity and specificity were calculated as explained in the Methods section, and the area under the ROC curve was used as a measure of performance. UCDB: prediction of drug candidates that can down-regulate genes up-regulated in cancer. DCUB: prediction of drug candidates that can up-regulate genes down-regulated in cancer. It shows that MFM consistently outperforms the two methods in different datasets and diseases

We compared the predictability of our method with that of the computational drug repositioning methods, which screen drugs based on the anti-correlation between similar gene and disease signatures, omitting the functional correlation between genes. In order to compare the performance with Shegimizu et al. [22], and CMap [20], we obtained the expression data of 1251, 1079 and 1182 compounds treated in MCF7, HL60 and PC3 from CMap data sets. We used methods to generate ranked drug lists and compared the highest AUC scores. As shown in Fig. 2 MFM consistently outperforms the 2 pervious methods, sometimes by wide margins.

#### Recall rate

Among 2587 bioactive compounds with FDR less than 0.05, 20/20 (*p* = 2.5E-4) FDA breast cancer drugs and 150/173 (*p* = 3.1E-10) clinical drugs (compounds that have been in clinical trials for breast cancer, Additional file 5) were recalled. For prostate cancer, among 1668 bioactive compounds with FDR less than 0.05, 10/11 (*p* = 2.6E-2) FDA prostate cancer drugs and 89/113 (*p* = 6.3E-6) clinical drugs were recalled. Significance was calculated using the Fisher exact test.

### Functional plausibility

#### Breast cancer

One way to characterize the functional implications of breast cancer MAGs is by estimating the chance probability of their observed distribution over KEGG pathways. We took the MAGs (MAG-UP, see, Additional file 6) that produced the drug ranked lists with the highest AUC scores after optimization. The MAGs contain 40 breast cancer mutations and their 237 filtered first nearest neighbors on the FLN, which are up regulated in breast cancer (see Additional file 6).

As shown in Additional file 6, we found 95 pathways over-represented in breast cancer (FDR < 0.05), 18 of which are classified in KEGG as cancer pathways (22 of the 287 KEGG pathways, are labeled cancer-related). For example, [28] found that the spliceosome assembly pathway is enriched in genes that are overexpressed in breast cancer samples, compared to benign lesions. They have shown that siRNA-mediated depletion of SmE (SNRPE) or SmD1 (SNRPD1) led to a marked reduction of cell viability in breast cancer cell lines, whereas it had little effect on the survival of the nonmalignant MCF10A breast epithelial cells [29].

In addition, signaling pathways that regulate pluripotent stems cells are enriched in overexpressed genes that are in the functional neighborhood of genes mutated in breast cancer tissue (MAGs, *p* = 4E-09). The deregulation of these pathways many play a role in the development of chemoresistance of cancer stem cells, including breast cancer [30]. Other published breast cancer causal pathways such as Estrogen signaling [31], ErbB [32], neurotrophin [33], MAPK [34] and PI3K/AKT [35] were significantly enriched in mutation associated genes (MAGs).

#### Prostate cancer

A similar approach was followed for prostate cancer. As summarized in Additional file 6, we found 117 enriched pathways (FDR <0.05), 18 of which are KEGG cancer pathways, including the prostate cancer pathway (*p* = 6.9E-10). There was also supporting evidence that showed deregulation of the enriched pathways in prostate cancer. For example, T cell infiltration of the prostate induced by androgen withdrawal has been found in patients with prostate cancer [36]; the androgen-androgen receptor (AR) system plays vital roles in prostate cancer development and progression [37]. Insulin-like growth factor 1 or insulin signaling has been found to activate androgen signaling through direct interactions of Foxo1 with androgen receptors. Intervention of IGF1/insulin-phosphatidylinositol 3-kinase-Akt signaling was reported to be of clinical value for prostate cancer. T cell receptor, PI3K-Akt, FoxO, and insulin signaling pathways were highly ranked candidates with *p* < E-05.

A number of studies have shown that breast and prostate cancer are genetically related [38, 39], as are almost all cancers to various degrees. Our finding that breast and prostate cancer share 80 pathways is a striking illustration of this connection (see Additional file 6). We expect that the selected drug candidates having a strong functional relation (mutual predictability score) with this set of genes could potentially correct these aberrant functions.

### MFM provides functional insight

We compared the functional information gained from MAGs with information obtained using disease differentially expressed genes (DEGs) (often referred to as disease signature genes) exclusively [19, 20]. As shown in Additional file 6, we found that our current method identifies more significantly enriched pathways and well-documented breast cancer and prostate cancer pathways than does the use of differential expression alone. To make a comparison, we mapped DEGs onto KEGG pathways. For breast cancer, one set contains the most up-regulated 247 DEGs; for prostate cancer, there were 333 up-regulated DEGs. The disease DEGs were generated from the expression data as explained in Transcript Level. These results taken collectively suggest that the inclusion of mutational and functional information into *disease gene signatures,* substantially improves prediction of disease mechanism and adds specificity and accuracy to the identification of repositioned candidates.

### Experimental validation

#### Repositioned drug candidates inhibit metabolism of breast cancer cells

We employed an MTT assay to assess cancer cell viability after treatments of 5 repositioned drug candidates (Table 2) [40]. In particular, we tested the viability of 2 breast cancer cell lines: MCF7 (Luminal A subtype), and SUM 149 (Triple negative, inflammatory breast cancer subtype). We assessed non specific drug toxicity by comparing the inhibition with that obtained against the immortalized but non-malignant MCF10A cell line.

**Fig. 3:**
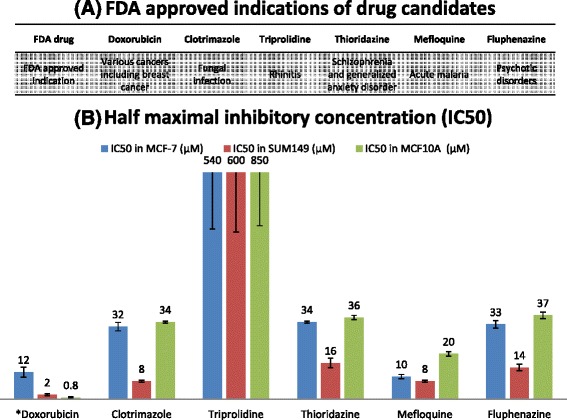
**a** FDA approved indications of predicted drug candidates; **b** Half maximal inhibitory concentration (IC50) (μM) of predicted drug candidates and Doxorubicin against MCF7, SUM149 and MCF10A; **c** and **d** Therapeutic index (TI) and maximal inhibitory concentrations (E_max_) of predicted repositioned drug candidates on MCF7, SUM149 and MCF10A. (*Currently used FDA drug for breast cancer; Therapeutic index (TI) was calculated as a ratio of the IC50 of MCF10A, to the IC50 of MCF7 and SUM149)

As shown in Additional file 7: Figure S2, Additional file 8: Figure S3, Additional file 9: Figure S4, Additional file 10: Figure S5, Additional file 11: Figure S6 and Additional file 12: Figure-S7, MCF7, SUM149 and MCF10A cells exposed to increasing concentrations of drugs for 24 h exhibited a dose dependent reduction in viability. The important measure of efficacy is therapeutic index (TI), the IC50 of a drug when it targets a non-tumor cell line, relative to its IC50 when it targets a tumor cell line. As shown in Fig. 3, the TIs of candidates tested against MCF7 and SUM149 are all substantially higher than that of Doxorubicin. In addition, all drug candidates except for Triprolidine achieved maximum efficacy (E_max_) at lower concentrations than did Doxorubicin.

## Discussion

We developed a computational drug screening method -- based on the correlation between functional modules of genes perturbed by diseases and drugs -- that could potentially accelerate the introduction of new therapeutics for serious diseases and conditions. Our approach performed substantially better than previous methods by computational measures, and successfully predicted novel drugs that had higher inhibitory effect against breast cancer in vitro than Doxorubicin. The study benefited substantially from LINCS, the most up to date drug response expression data sets currently available.

A number of computational drug-repositioning methods that utilized CMap have been devised and the efficacy of identified drugs have been supported by in vivo [16, 19] experiments. However, the methodologies are exclusively based on gene expression, without taking disease driver/mutated genes or functional information between genes into account. Sirota, M., et al. [15] searched for drug candidates based on similarities between drug response gene signatures (DEG) and [12] predicted drug molecular functions based on drug response gene signatures.

Here we indicate a method that has taken this into account and shows better performance than previous methods that utilized solely DEGs. We also showed that there was more functional information gained from MAGs than significantly differentially expressed genes (DEGs). Therefore, we believe that the method could screen more effective therapeutics than previous methods.

Of the five drugs for which we did preliminary in vitro tests, they all have higher TI in both cell types than does Doxorubicin. Mefloquine is a lipophilic molecule that is an FDA-approved anti-malaria agent. It has 3 known protein targets: Fe(II)-protoporphyrin IX, hemoglobin subunit alpha, and A2A adenosine receptor (A2AR). Its antimalarial action is believed to result from inhibition of heme polymerization within the food vacuole in the blood stages of the malaria life cycle [41]. Its potential role as a cancer therapeutic; however, stems from its antagonistic action on A2AR [42].

A study has shown that antagonizing A2AR could provide a basis for cancer immunotherapy [43]. Preclinical studies have confirmed that blockade of A2a receptor activation has the ability to markedly enhance anti-tumor immunity and be effective against melanoma and lymphoma [44–46].

Tumors may evade immune repose by usurping pathways; such as adenosinergic signaling pathway, that negatively regulates immune response. Tumors and its microenvironment have been found to have high levels of adenosine and ATP, which is triggered by increased cellular turnover and hypoxia [43]. The extracellular adenosine then activates specific purinergic receptors such as A2AR. The activation of A2AR in cancer results in inhibition of the immune response to tumors via suppression of T regulatory cell function and inhibition of natural killer cell cytotoxicity and tumor-specific CD4^+^ and CD8^+^ T cell activity, therefore, inhibition of A2AR by specific antagonists may enhance anti-tumor immunity.

Immunosuppression is associated with hypoxia and accelerated cell turn over. In accordance with the findings, in our analysis of pathway enrichment of MAGs for breast cancer, cell cycle, HIF1 and T cell signaling pathways were significantly dysregulated in breast cancer. Therefore, Mefloquine, the A2aR antagonist could be applied as an effective immunotherapeutic strategy.

Fluphenazine and Thioridazine are both antipsychotics. The mechanism of action of fluphenazine is not well established, but it is known to antagonize dopamine by binding to the D2 receptor. Thioridazine binds a range of receptor types including dopamine and various serotonin receptor subtypes. The relationship to inhibition of transformed (MCF7 and SUM149) cells is not entirely obvious.

In our in vitro study, breast cancer cells (MCF7, SUM149 and MCF10A) had shown resistance against Doxorubicin. The Emax of Doxorubcin was higher than 4 out of 5 of our candidate drugs, which corresponds with the reported fact that breast cancer patients show drug resistance against Doxorubicin. It also suggests the ability of our drug candidate to overcome the drug resistance. The study [47] has found that Thioridazine antagonized dopamine receptors, which are expressed on cancer stem cells (CSC) and breast cancer cells, and could induce death of leukemia cancer stem cells preferentially without harming normal blood stem cells. The dopamine receptor pathway is known to regulate the growth of CSCs [48]. Therefore, Fluphnazine and Thioridazine could inhibit drug resistance of breast cancers by modulating CSC through dopamine receptor signaling pathway.

## Conclusion

MFM, which utilizes a functional-linkage network, known mutations, and altered RNA levels, appears to be a promising method for identifying multi-targeted drug candidates that can correct aberrant cellular functions. In particular the computational performance exceeded that of other CMap-based methods, and in vitro experiments indicate that 5/5 candidates have therapeutic indices superior to that of Doxorubicin in MCF7 and SUM149 cancer cell lines. This new approach has the potential to provide a more efficient drug discovery pipeline.

## Abbreviations

A2AR, adenosine A2a receptor; AUC, area under the curve; CMap, connectivity map; CSC, cancer stem cells; DCUB, down regulated cancer genes up regulated bioactive compounds; DEG, differentially expressed genes; DMSO, Dimethylsulfoxide; DNA, deoxyribonucleic acid; DRG, drug response gene; EMax, maximal inhibitory concentration; FDA, Food and drug administration; FDR, false discovery rate; FLN, functional linkage network; GEO, gene expression omnibus; IC50, half maximal inhibitory concentration; KEGG, Kyoto encyclopedia of genes and genomes; LINCS, library of integrated network based cellular signatures; MAG, mutation associated gene; MFM, method of functional modules; MP, mutual predictability; MTT, 3-(4,5-Dimethylthiazol-2-Yl)-2,5-Diphenyltetrazolium Bromide; OMIM, online mendelian inheritance in man; RNA, ribonucleic acid; ROC, receiver operating characteristic; TCGA, the cancer genome atlas; TI, therapeutic index; UCDB, up regulated cancer genes down regulated bioactive compounds

## Additional files

Additional file 1: is a table listing well-documented mutated genes for breast cancer, prostate cancer and leukemia. (XLS 889 kb) Additional file 2: and 2–1 show detailed process of MP score computation. (DOCX 106 kb) Additional file 3: Figure S1. An example of mutual predictability score computation. For ROC curve M-D (sensitivity plotted against 1-specificity), sensitivity and 1 – specificity are defined as follows: sensitivity = TP / (TP + FN), 1 - specificity = FP / (TN + FP), where TP is the number of DRG genes above a particular Si cutoff, TN is the number of genes associated with neither disease below the cutoff, FP is the number of genes associated with neither disease above the cutoff, and FN is the number of DRG genes below the cutoff. ROC curve D-M was plotted in the same way. The MP score (0.73) is defined as the geometric mean of area under the ROC M-D and ROC D-M curves: AUC M-D (0.81) and AUCD-M (0.65). (PPTX 299 kb) Additional file 4: shows detailed description of identified drug candidates for breast and prostate cancer. (XLSX 140 kb) Additional file 5: lists FDA-approved and clinical drugs for breast and prostate cancer. (XLSX 52 kb) Additional file 6: is a table listing MAGs for breast cancer and prostate cancer, also has the listing enriched KEGG pathways in breast cancer and prostate cancer. (XLSX 46 kb) Additional file 7: Figure S2. Titration curves of cell viability under treatment of Doxorubicin. Viability of MCF10A, MCF7 and SUM 149 cells exposed to Doxorubicin with concentrations ranging from 0.5 μM to 200 μM after 24 h incubation. The relative viability was calculated as relative viability = (experimental absorbance - background absorbance)/ (absorbance of untreated controls - background absorbance of untreated controls) × 100 % (means ± SD, n = 6). (PPTX 53 kb) Additional file 8: Figure S3. Titration curves of cell viability under treatment of Mefloquine. Viability of MCF10A, MCF7 and SUM 149 cells exposed to Mefloquine with concentrations ranging from 3.125 μM to 100 μM after 24 h incubation. The relative viability was calculated as relative viability = (experimental absorbance - background absorbance)/ (absorbance of untreated controls - background absorbance of untreated controls) × 100 % (means ± SD, n = 3). (PPTX 53 kb) Additional file 9: Figure S4. Titration curves of cell viability under treatment of Clotrimazole. Viability of MCF10A, MCF7 and SUM 149 cells exposed to Clotrimazole with concentrations ranging from 3.125 μM to 100 μM after 24 h incubation. The relative viability was calculated as relative viability = (experimental absorbance - background absorbance)/ (absorbance of untreated controls - background absorbance of untreated controls) × 100 % (means ± SD, n = 3). (PPTX 53 kb) Additional file 10: Figure S5 Titration curves of cell viability under treatment of Thioridazine. Viability of MCF10A, MCF7 and SUM 149 cells exposed to Thioridazine with concentrations ranging from 3.125 μM to 100 μM after 24 h incubation. The relative viability was calculated as relative viability = (experimental absorbance - background absorbance)/ (absorbance of untreated controls - background absorbance of untreated controls) × 100 % (means ± SD, n = 3). (PPTX 54 kb) Additional file 11: Figure S6. Titration curves of cell viability under treatment of Fluphenazine. Viability of MCF10A, MCF7 and SUM 149 cells exposed to Fluphenazine with concentrations ranging from 3.125 μM to 100 μM after 24 h incubation. The relative viability was calculated as relative viability = (experimental absorbance - background absorbance)/ (absorbance of untreated controls - background absorbance of untreated controls) × 100 % (means ± SD, n = 3). (PPTX 55 kb) Additional file 12: Figure S7. Titration curves of cell viability under treatment of Triprolidine. Viability of MCF10A, MCF7 and SUM 149 cells exposed to Triprolidine with concentrations ranging from 31.25 μM to 1000 μM after 24 h incubation. The relative viability was calculated as relative viability = (experimental absorbance - background absorbance)/ (absorbance of untreated controls - background absorbance of untreated controls) × 100 % (means ± SD, n = 3). (PPTX 55 kb) Additional file 13: is a table listing predicted drug candidates for breast and prostate cancer using LINCS dataset. (XLSX 1152 kb)
