# Supplementary material for: A network based approach to drug repositioning identifies plausible candidates for breast cancer and prostate cancer
Source: BMC Med Genomics. 2016 Jul 30;9:51. doi: 10.1186/s12920-016-0212-7 (PMC4967295; doi:10.1186/s12920-016-0212-7)
Supplement: Additional file 2: — and 2–1 show detailed process of MP score computation. (DOCX 106 kb) [file 12920_2016_212_MOESM2_ESM.docx]

**Mutual predictability (MP) score computation example**

The following is the computation process of MP scores (M-D and D-M) using 362 breast cancer MAG genes as seed genes (AUC _M-D_) and 700 DRG genes of the drug (topotecan, CPC011_MCF7_6H_X1_B3_DUO52HI53LO_B07, from LINCS) as seed genes (AUC _D-M_) (genes are listed in **Additional file 2-1**). The steps are as follow:

Step 1: Score and rank each gene connected to a seed using the disease mutual predictability score *S_i_*:

$$S_{i}=\sum_{j\in seeds} w_{ij}$$

where w*_ij_* weights the link between gene *i* and seed *j, and* the score is 0 if there is no seed connection. In addition, DRG genes that overlap with the seed genes from MAG are given a *S_i_* score of infinity and ranked on the top. The ranked list of connected genes is listed in **Additional file 2-1**.

Step 2: We then generate an ROC (sensitivity plotted against 1- specificity) and compute the area under the curve (AUC_M-D_ = 0.81, **Figure S1**). Sensitivity and (1- specificity) are defined as follow:

Sensitivity = TP / (TP +FN)

1 – Specificity = FP / (TN + FP)

where TP is the number of DRG genes above a particular *S_i_* cutoff, TN is the number of genes associated with neither disease below the cutoff, FP is the number of genes associated with neither disease above the cutoff, and FN is the number of DRG genes below the cutoff

Step 3: Similarly, we can calculate AUC _D-M_ = 0.65 (**Figure S1**). The MP score is defined as the geometric mean of AUC _M-D_ and AUC _D-M:_

Mutual Predictability (MAG and DRG) = $\sqrt{\mathrm{AUC}D-M \times AUC M-D}$ = 0.73
