# Supplementary figures and images for: A network based approach to drug repositioning identifies plausible candidates for breast cancer and prostate cancer
Source: BMC Med Genomics. 2016 Jul 30;9:51. doi: 10.1186/s12920-016-0212-7 (PMC4967295; doi:10.1186/s12920-016-0212-7)

## Slide 1
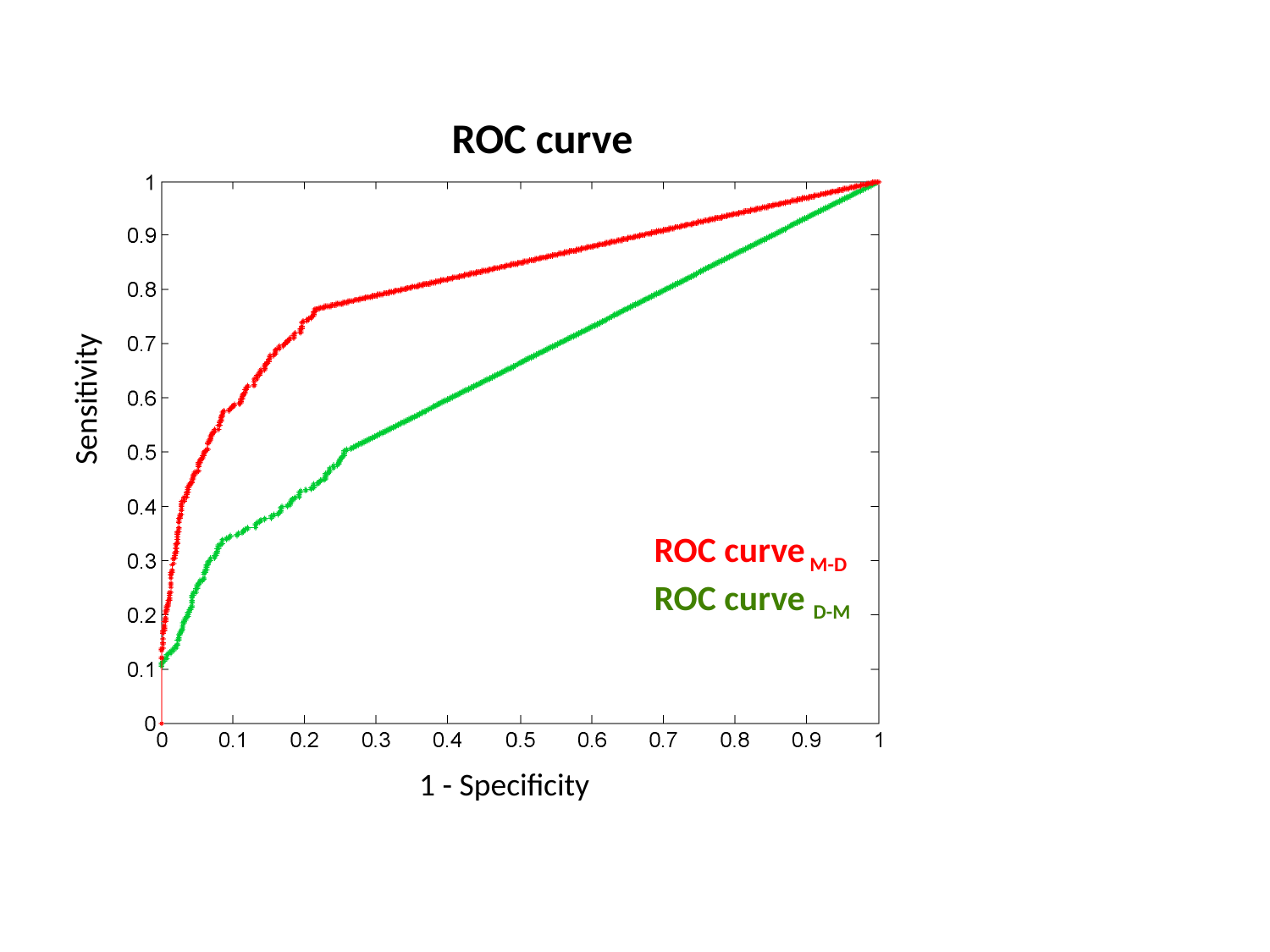

ROC curve
Sensitivity
ROC curve M-D
ROC curve D-M
1 - Specificity

Supplement: Additional file 3: Figure S1. — An example of mutual predictability score computation. For ROC curve M-D (sensitivity plotted against 1-specificity), sensitivity and 1 – specificity are defined as follows: sensitivity = TP / (TP + FN), 1 - specificity = FP / (TN + FP), where TP is the number of DRG genes above a particular Si cutoff, TN is the number of genes associated with neither disease below the cutoff, FP is the number of genes associated with neither disease above the cutoff, and FN is the number of DRG genes below the cutoff. ROC curve D-M was plotted in the same way. The MP score (0.73) is defined as the geometric mean of area under the ROC M-D and ROC D-M curves: AUC M-D (0.81) and AUCD-M (0.65). (PPTX 299 kb) [file 12920_2016_212_MOESM3_ESM.pptx]

## Slide 1
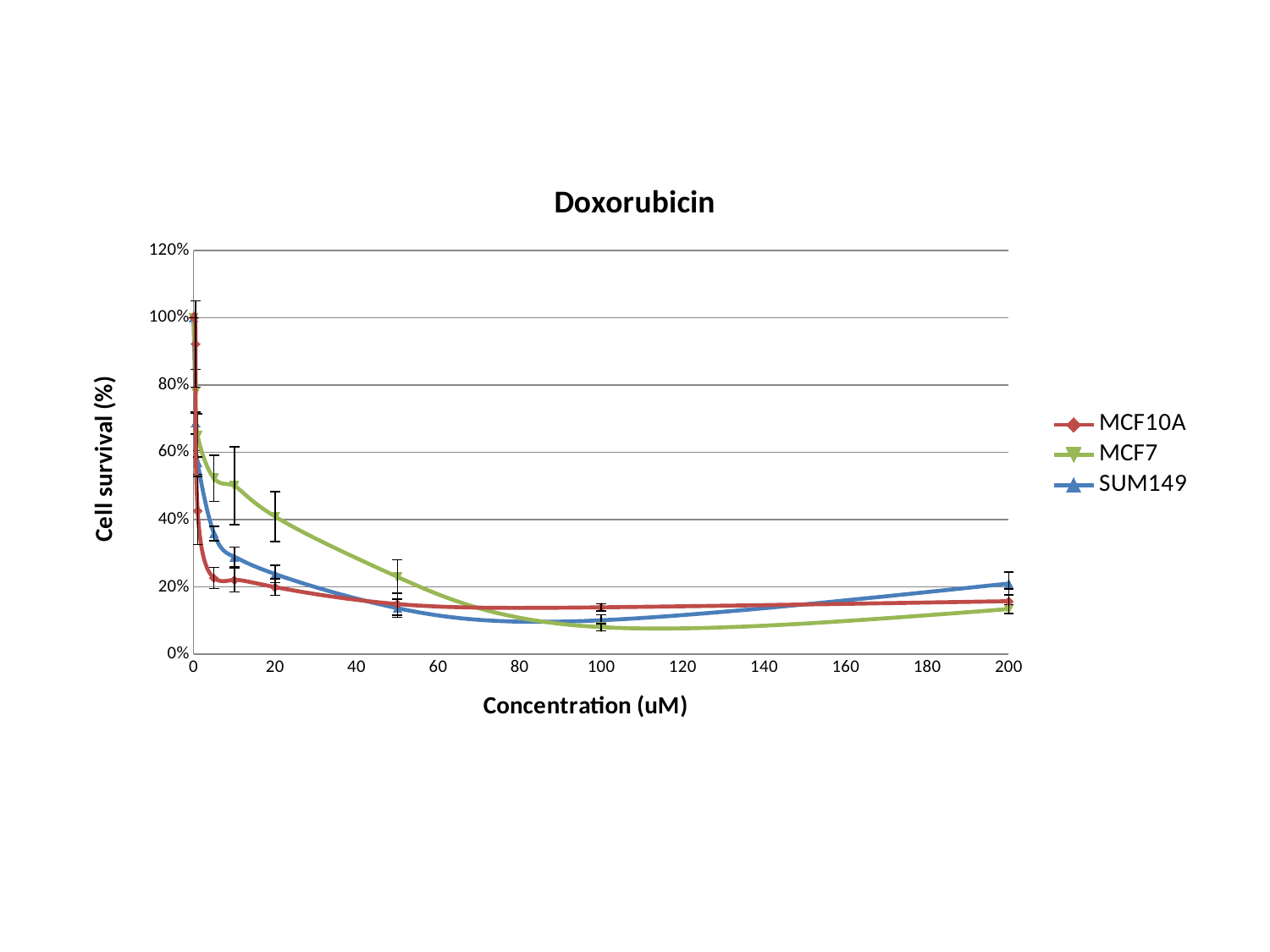

#
### Chart: Doxorubicin
| Category | | | |
|---|---|---|---|

Supplement: Additional file 7: Figure S2. — Titration curves of cell viability under treatment of Doxorubicin. Viability of MCF10A, MCF7 and SUM 149 cells exposed to Doxorubicin with concentrations ranging from 0.5 μM to 200 μM after 24 h incubation. The relative viability was calculated as relative viability = (experimental absorbance - background absorbance)/ (absorbance of untreated controls - background absorbance of untreated controls) × 100 % (means ± SD, n = 6). (PPTX 53 kb) [file 12920_2016_212_MOESM7_ESM.pptx]

## Slide 1
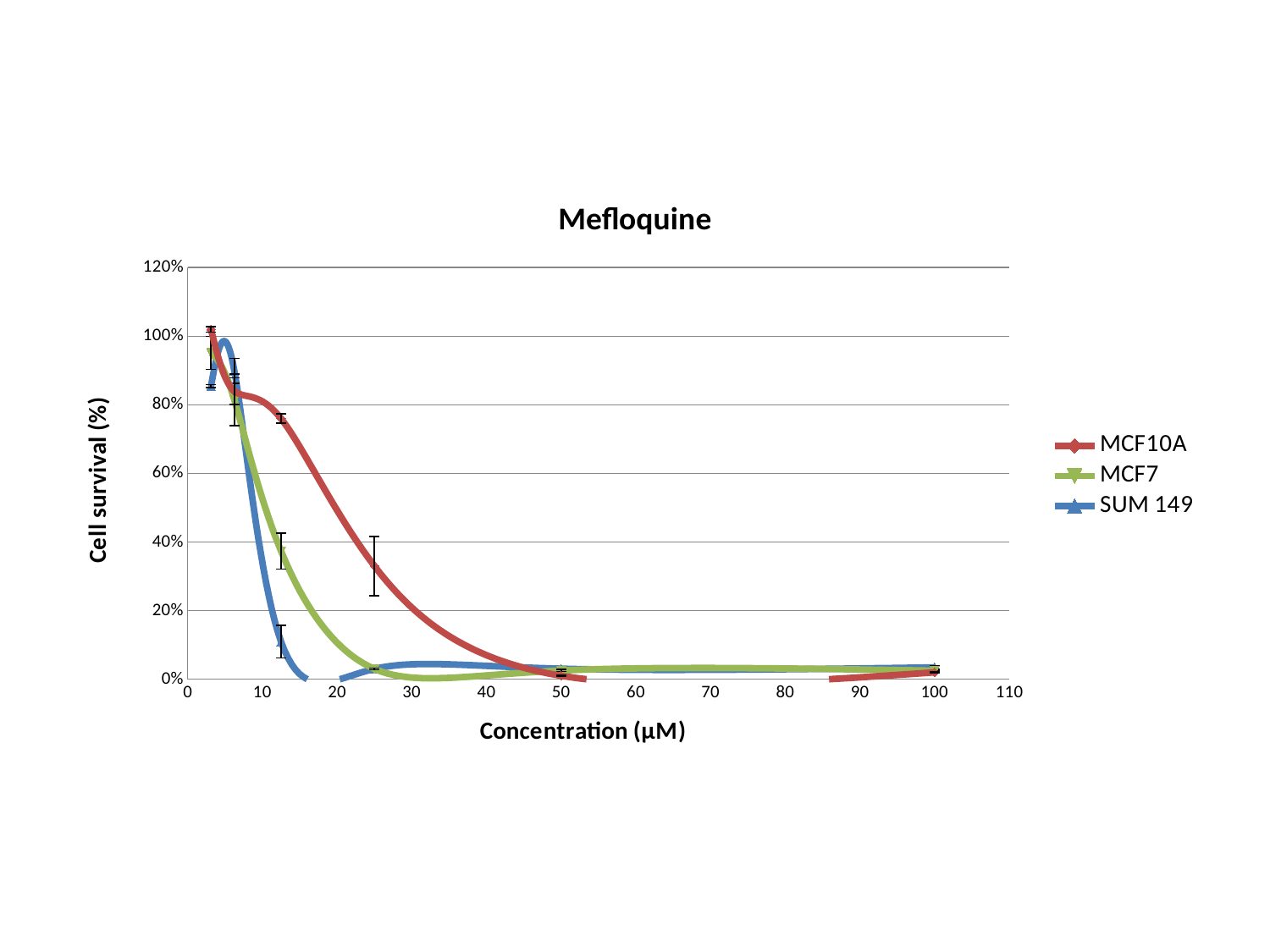

#
### Chart: Mefloquine
| Category | | | |
|---|---|---|---|

Supplement: Additional file 8: Figure S3. — Titration curves of cell viability under treatment of Mefloquine. Viability of MCF10A, MCF7 and SUM 149 cells exposed to Mefloquine with concentrations ranging from 3.125 μM to 100 μM after 24 h incubation. The relative viability was calculated as relative viability = (experimental absorbance - background absorbance)/ (absorbance of untreated controls - background absorbance of untreated controls) × 100 % (means ± SD, n = 3). (PPTX 53 kb) [file 12920_2016_212_MOESM8_ESM.pptx]

## Slide 1
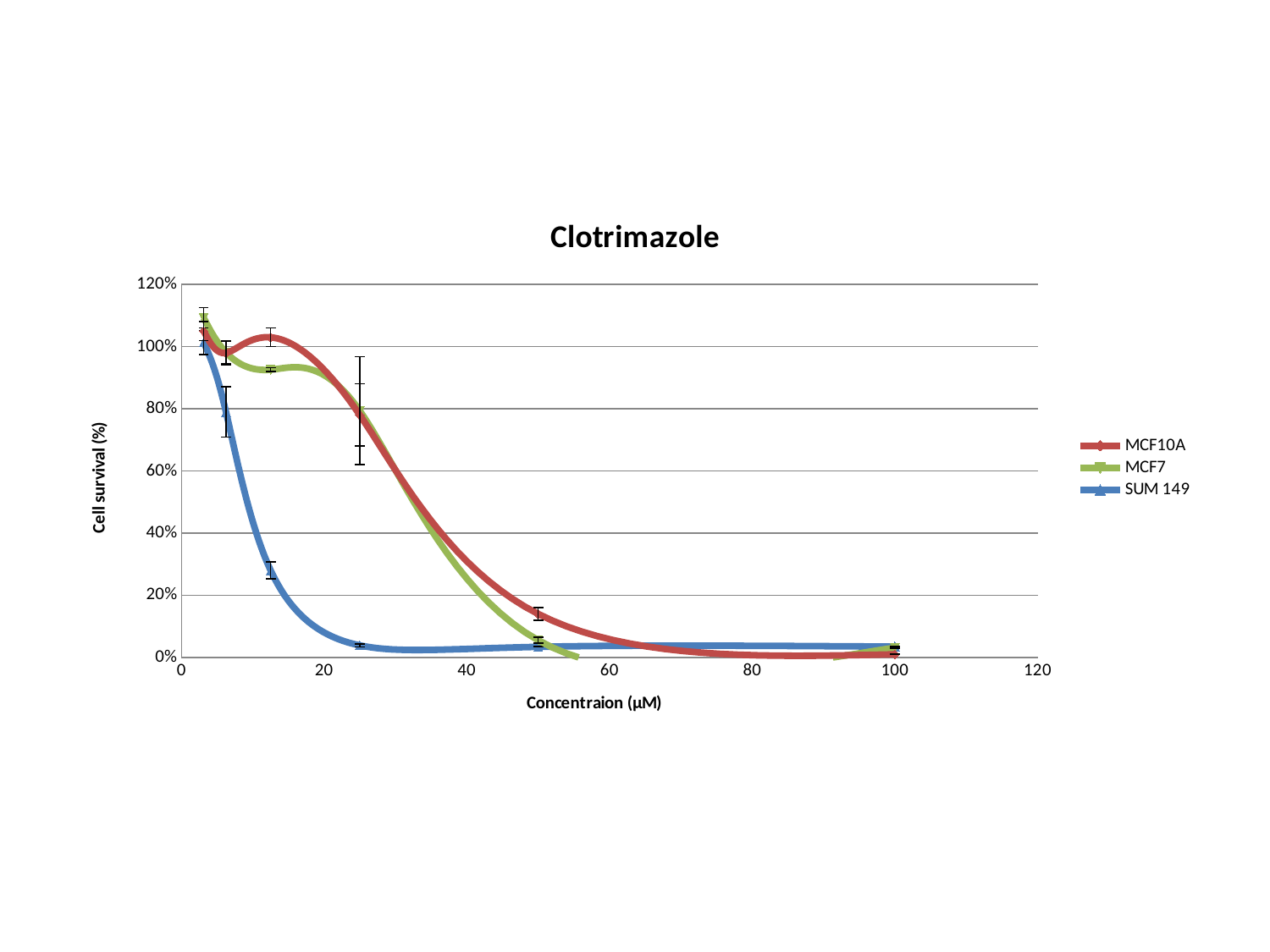

#
### Chart: Clotrimazole
| Category | | | |
|---|---|---|---|

Supplement: Additional file 9: Figure S4. — Titration curves of cell viability under treatment of Clotrimazole. Viability of MCF10A, MCF7 and SUM 149 cells exposed to Clotrimazole with concentrations ranging from 3.125 μM to 100 μM after 24 h incubation. The relative viability was calculated as relative viability = (experimental absorbance - background absorbance)/ (absorbance of untreated controls - background absorbance of untreated controls) × 100 % (means ± SD, n = 3). (PPTX 53 kb) [file 12920_2016_212_MOESM9_ESM.pptx]

## Slide 1
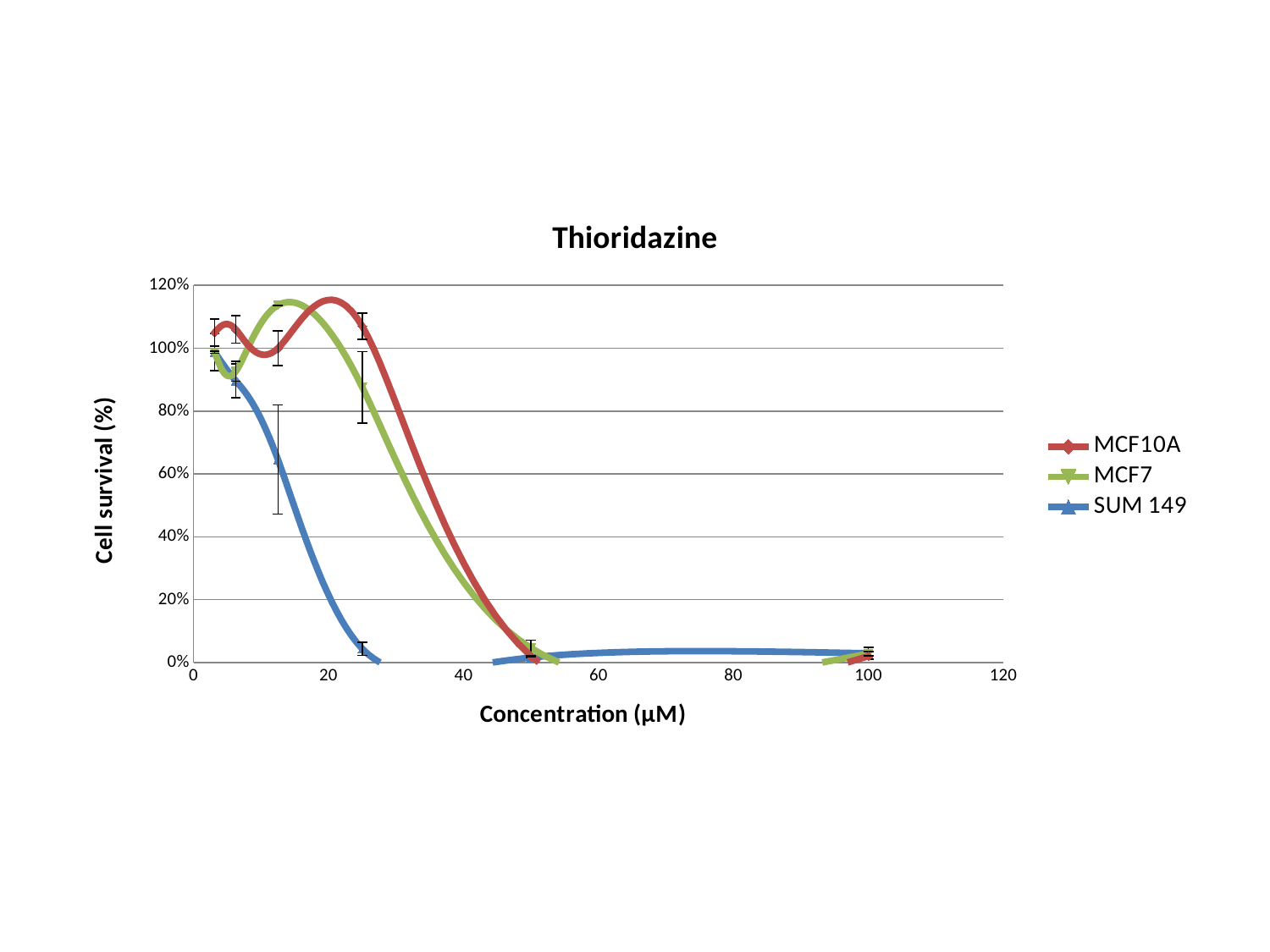

#
### Chart: Thioridazine
| Category | | | |
|---|---|---|---|

Supplement: Additional file 10: Figure S5 — Titration curves of cell viability under treatment of Thioridazine. Viability of MCF10A, MCF7 and SUM 149 cells exposed to Thioridazine with concentrations ranging from 3.125 μM to 100 μM after 24 h incubation. The relative viability was calculated as relative viability = (experimental absorbance - background absorbance)/ (absorbance of untreated controls - background absorbance of untreated controls) × 100 % (means ± SD, n = 3). (PPTX 54 kb) [file 12920_2016_212_MOESM10_ESM.pptx]

## Slide 1
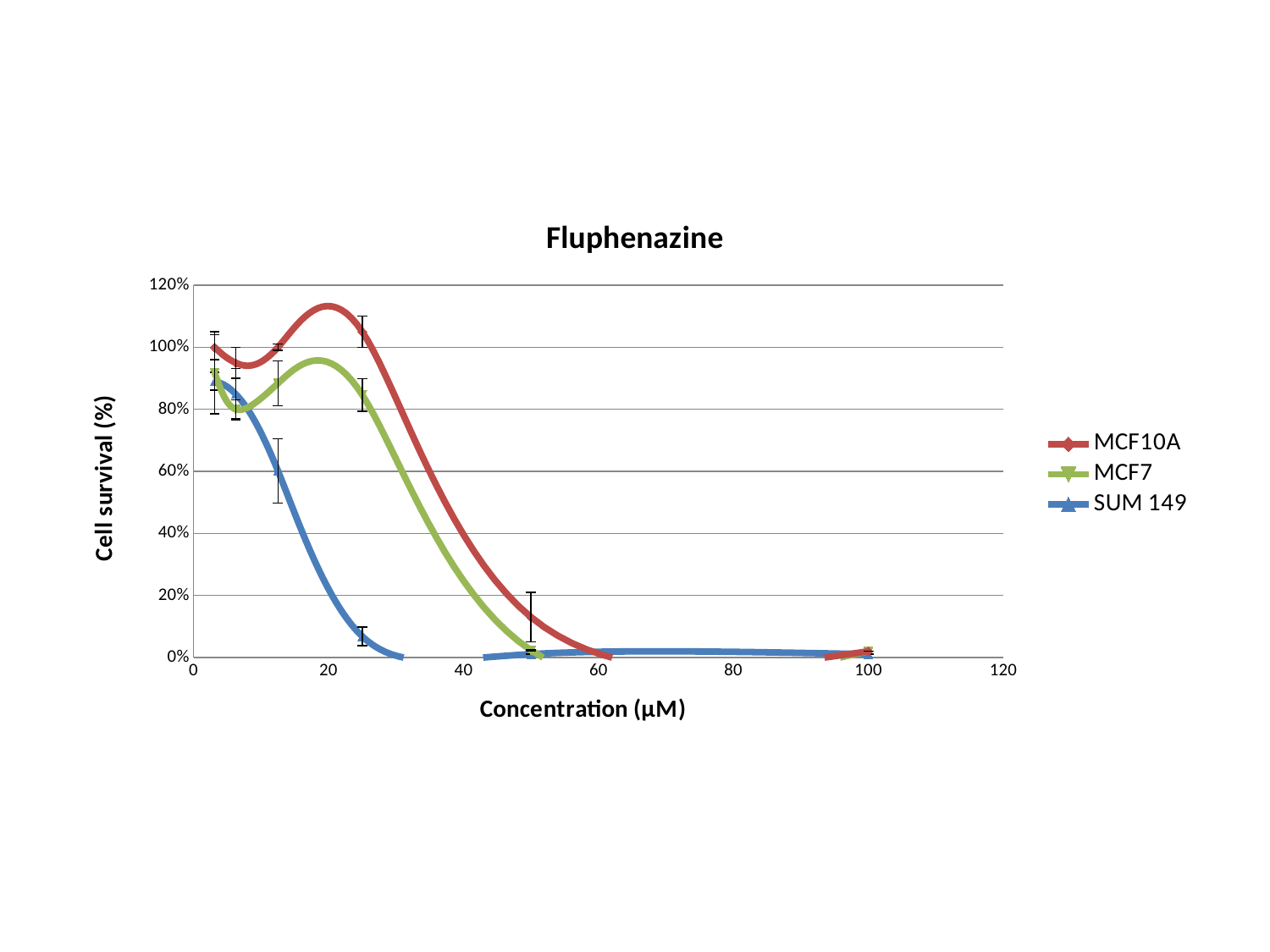

#
### Chart: Fluphenazine
| Category | | | |
|---|---|---|---|

Supplement: Additional file 11: Figure S6. — Titration curves of cell viability under treatment of Fluphenazine. Viability of MCF10A, MCF7 and SUM 149 cells exposed to Fluphenazine with concentrations ranging from 3.125 μM to 100 μM after 24 h incubation. The relative viability was calculated as relative viability = (experimental absorbance - background absorbance)/ (absorbance of untreated controls - background absorbance of untreated controls) × 100 % (means ± SD, n = 3). (PPTX 55 kb) [file 12920_2016_212_MOESM11_ESM.pptx]

## Slide 1
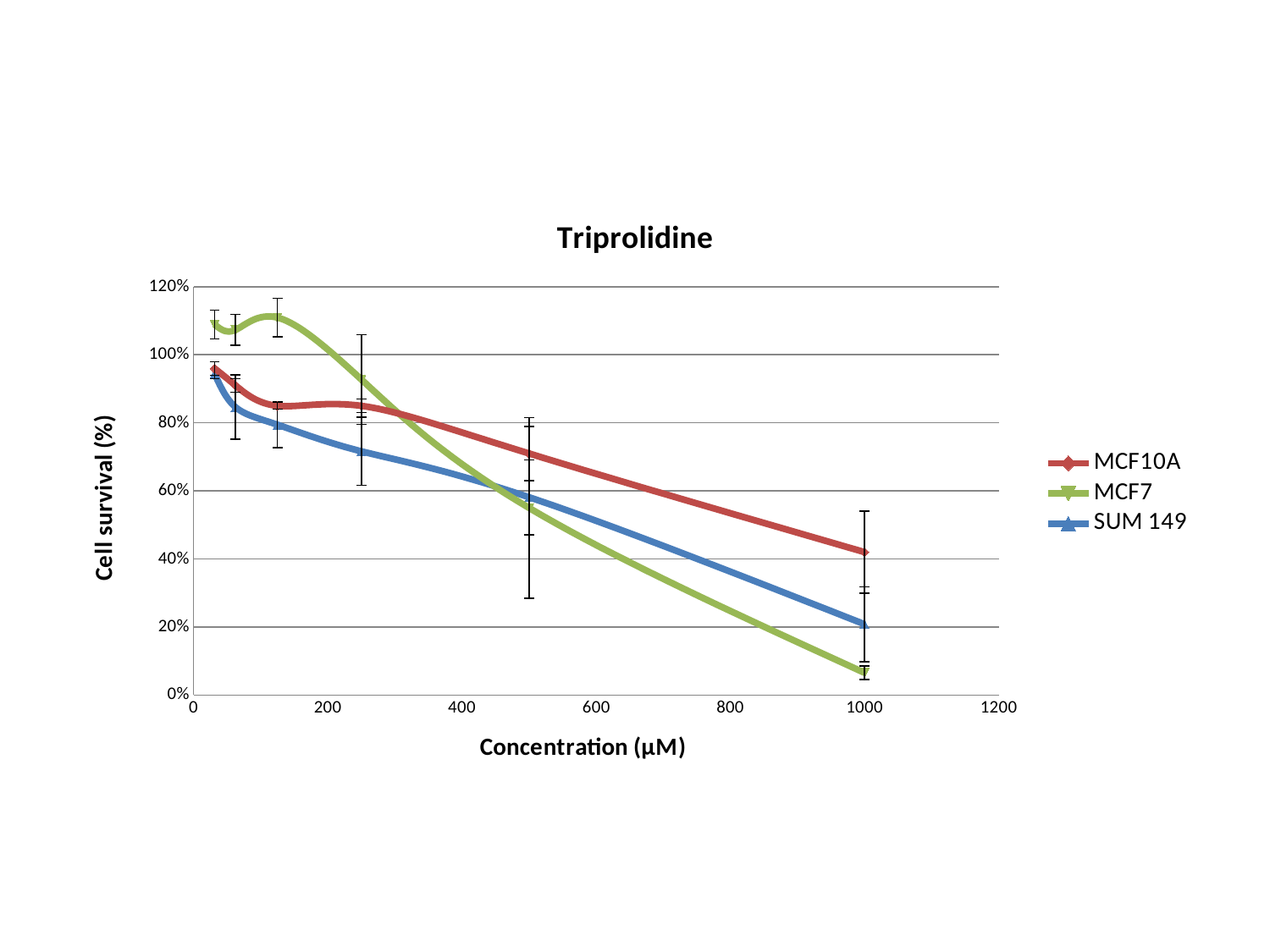

#
### Chart: Triprolidine
| Category | | | |
|---|---|---|---|

Supplement: Additional file 12: Figure S7. — Titration curves of cell viability under treatment of Triprolidine. Viability of MCF10A, MCF7 and SUM 149 cells exposed to Triprolidine with concentrations ranging from 31.25 μM to 1000 μM after 24 h incubation. The relative viability was calculated as relative viability = (experimental absorbance - background absorbance)/ (absorbance of untreated controls - background absorbance of untreated controls) × 100 % (means ± SD, n = 3). (PPTX 55 kb) [file 12920_2016_212_MOESM12_ESM.pptx]
